# Evaluation of a medical–rehabilitation integration model to improve rehabilitation service efficiency in a tertiary rehabilitation hospital

**DOI:** 10.1515/med-2026-1482

**Published:** 2026-08-03

**Authors:** Lin Chen, Qi Miao, Yu-Tong Jiang, Hong-Jian Lu

**Affiliations:** College of Public Health, Nantong University, Nantong, China; Department of Procurement Management, Nantong Rehabilitation Hospital, Nantong, China; Department of Public Health and Preventive Medicine, Nantong Rehabilitation Hospital, Nantong, China; College of Business, Nantong University, Nantong, China; Department of Hospital Administration, Nantong First People’s Hospital, Nantong, China

**Keywords:** medical‒rehabilitation integration, multidisciplinary collaboration, rehabilitation hospital, service system construction

## Abstract

**Objectives:**

To evaluate the association of the medical‒rehabilitation services in a tertiary rehabilitation hospital through the implementation of a medical–rehabilitation integration model.

**Methods:**

Nantong Rehabilitation Hospital was used as a representative case. The effects of implementing the medical–rehabilitation integration model on medical service delivery models, medical resource utilization, and the operational efficiency of major disease categories were evaluated using comparative data analysis and other analytical approaches.

**Results:**

Following implementation of the medical–rehabilitation integration model, which encompassed acute-phase management, early rehabilitation intervention, the rehabilitation phase, and long-term management, continuity across all stages of diagnosis and treatment was achieved. This integrated approach addressed patients’ comprehensive and individualized needs related to diagnosis, treatment, and rehabilitation. Indicators of medical resource utilization and the operational efficiency of major disease categories showed significant improvement after implementation.

**Conclusions:**

The medical–rehabilitation integration model was associated with enhanced medical resource utilization, optimized the operational efficiency of disease categories, and improved patient satisfaction through multidisciplinary collaboration, early rehabilitation intervention, information support, and process optimization. Strengthening the three-tier rehabilitation service chain, reinforcing the integrated development of clinical practice, education, and research, and ensuring sustained policy and resource support are necessary to promote the long-term development of the medical–rehabilitation integration model.

## Introduction

In 2016, the United Nations General Assembly adopted the 2030 sustainable development goals (SDGs), within which universal health coverage places urgent emphasis on strengthening rehabilitation service capacity [1]. China’s demographic structure has been undergoing substantial change, characterized by accelerated population aging and a high prevalence of chronic diseases, thereby imposing new demands on the medical and health service system. Conventional medical service models have remained primarily focused on diagnosis and treatment during the acute phase of illness, with relatively limited attention directed toward functional recovery and quality of life in subsequent stages. This imbalance has resulted in fragmentation of the treatment–rehabilitation continuum and has restricted patients’ access to continuous and systematic rehabilitation support.

Within this context, rehabilitation medicine has assumed increasing importance, and the effective integration of rehabilitation concepts and technologies across the entire clinical diagnostic and therapeutic process, with coordinated alignment between medical treatment and rehabilitation, has emerged as a key determinant of healthcare quality. Representative approaches include enhanced recovery after surgery, pre-rehabilitation, early rehabilitation, integrated early rehabilitation, and clinical rehabilitation integration [2]. At the international level, medical–rehabilitation integration models commonly incorporate multimodal rehabilitation strategies, including physical therapy, psychotherapy, and speech therapy, to establish comprehensive rehabilitation systems. Evidence from European studies indicates that such models provide substantial value for long-term patient health management, particularly in neurorehabilitation and geriatric rehabilitation.

In 2021, the Chinese Association of Rehabilitation Medicine formally introduced the medical–rehabilitation integration framework, which emphasizes patient-centered care, multidisciplinary collaboration, early intervention, and full-cycle service delivery [3]. The medical–rehabilitation integration model typically incorporates multiple collaborative mechanisms, including bidirectional referral pathways, optimization of the rehabilitation service chain, and information support systems [4]. Through the integration of medical resources, service accessibility is enhanced, redundant interventions are reduced, and more rational resource allocation and refined patient health management are achieved [5].

As regional hubs and technical centers for rehabilitation services, tertiary rehabilitation hospitals assume primary responsibility for the management of complex rehabilitation cases, the provision of technical guidance, and the coordination of regional resources. These institutions represent optimal platforms and critical nodes for advancing the medical–rehabilitation integration model.

Based on practical experience from a tertiary rehabilitation hospital in Nantong city, the present work provides a comprehensive evaluation of the medical–rehabilitation integration model through a systematic literature review and in-depth case analysis, with the objective of offering theoretical insights and practical guidance for model development and optimization in comparable institutions.

## Methods

### Study design

This investigation was conducted as a retrospective, single-center empirical study. Data were obtained from all patients discharged from Nantong Rehabilitation Hospital between January 1, 2022, and December 31, 2023. This study adopted retrospective data, which were fully anonymized aggregated population data rather than individual-level data. This study does not involve the ethics.

Nantong Rehabilitation Hospital is a public tertiary rehabilitation institution that was restructured from a secondary class A general hospital. The implementation of the medical–rehabilitation integration model progressed through three successive phases, including a rehabilitation department-led phase, a rehabilitation department intervention phase, and a medical–rehabilitation integration phase, which made the institution a representative example of this developmental pathway.

To evaluate the practical impact of the medical–rehabilitation integration model, medical resource utilization was comparatively analyzed between periods before and after implementation of the model. Statistical information extracted from institutional databases, including the hospital information system and the electronic medical record system, was used to assess key indicators. These indicators included bed occupancy rate, bed turnover rate, average length of stay, utilization rates of major rehabilitation equipment, medical staff workload, and the efficiency of human resource allocation.

### Specific practices of the medical–rehabilitation integration model

From the perspective of driving forces, the medical–rehabilitation integration model developed primarily in response to institutional needs related to survival and sustainable development, in combination with policy guidance and governmental promotion. During the initial stage, as the demand for rehabilitation services increased, a rehabilitation department was established in accordance with the hospital’s disciplinary development strategy to address the rehabilitation needs of selected patient populations and to enhance overall service capacity. At this stage, the rehabilitation department functioned in a relatively independent manner.

With advances in medical technology and progressive improvement in patient awareness of rehabilitation, clinical departments increasingly required rehabilitation involvement at specific stages of diagnosis and treatment to reduce disease-related complications and support functional recovery. Consequently, rehabilitation services shifted from isolated operation toward partial collaboration with clinical departments. As population aging intensified and the prevalence of chronic diseases continued to increase, the demand for rehabilitation services expanded substantially. Under these circumstances, the traditional model, which emphasized treatment with limited attention to rehabilitation, was insufficient to meet expectations for high-quality rehabilitation care. Concurrently, conceptual shifts reinforced the recognition that rehabilitation should be incorporated throughout the entire continuum of medical care.

From an organizational structure perspective, under the rehabilitation department–centric model, the rehabilitation department operated as an independent unit with its own medical staff and maintained relative structural separation from other departments, resulting in incomplete mechanisms for interdepartmental collaboration. Within the rehabilitation department intervention model, communication channels with clinical departments were partially established, and collaboration was conducted through consultations. However, a fully integrated organizational framework was not achieved, and the rehabilitation department continued to function primarily in a supportive role. Under the medical–rehabilitation integration model, the rehabilitation department assumed a central role through the establishment of multidisciplinary joint diagnosis and treatment teams involving multiple clinical departments. Rehabilitation physicians were paired with clinical physicians, departmental boundaries were reduced, and a highly integrated organizational structure was formed.

From the perspective of service content, the scope of rehabilitation services was further expanded. In the rehabilitation department-centric model, services were largely limited to rehabilitation interventions, such as limb function training and physical therapy, provided to patients after clinical stabilization and subsequent discharge or transfer to the rehabilitation department. These services were predominantly confined to the rehabilitation department, with limited coordination with clinical units.

The rehabilitation department intervention model introduced rehabilitation involvement during clinical care when indicated. For example, joint mobility training was provided to orthopedic patients after initial wound healing, reflecting a gradual transition toward the integration of rehabilitation with clinical treatment. Within the medical–rehabilitation integration model, rehabilitation assessment was initiated at admission and maintained throughout the entire course of treatment, including acute-phase bedside rehabilitation and immediate postoperative interventions. For instance, patients with stroke received swallowing function training concurrently with thrombolytic therapy in the neurology department, while nutritional support, psychological interventions, and related services were also incorporated (Table 1).

**Table 1:** Comparison of rehabilitation service delivery models across different implementation stages at Nantong Rehabilitation Hospital.

Dimension	Rehabilitation department–led	Rehabilitation department intervention	Medical–rehabilitation integration
Driving factors	Based on internal disciplinary development and hospital growth needs	Advances in medical technology and improved patient awareness of rehabilitation	Traditional “treatment-focused, rehabilitation-neglected” model no longer meets current demands; conceptual transformation is required
Organizational structure	Rehabilitation department functions as an independent unit with its own medical and nursing staff	No tightly integrated organizational structure; rehabilitation department remains in a supportive role	Multidisciplinary joint diagnosis and treatment teams established with the rehabilitation department as the core
Service content	Rehabilitation services mainly provided to patients transferred to the rehabilitation department after discharge or clinical stabilization	Rehabilitation department intervenes as needed during clinical treatment	Rehabilitation assessment and treatment initiated at admission and continued throughout the entire diagnostic and therapeutic process
Model characteristics	Independence	Limited level of coordination	Holism and continuity

Based on the above evolution, the medical–rehabilitation integration model implemented at Nantong Rehabilitation Hospital comprises a set of standardized core components that can be replicated in other settings. These include an organizational structure known as the “One Center, Three Departments” framework, multidisciplinary team (MDT) collaboration, early rehabilitation intervention, individualized rehabilitation planning, diversified service content (e.g., physical, psychological, and nutritional interventions), full-cycle information support, performance incentive mechanisms, and a quality control system.

### Definition of outcome measures

The following outcome measures were used to evaluate the effectiveness of the medical–rehabilitation integration model. Raw data for all indicators were extracted from the hospital information system (HIS), including medical record homepages, hospitalization cost records, and bed occupancy records.

Cure rate referred to the proportion of discharged patients in whom the disease was cured during the reporting period. Cure was defined as complete resolution of symptoms and full functional recovery, as determined by clinical physicians and recorded on the medical record homepage at discharge. Specific criteria varied by disease but generally included: (1) complete disappearance of lesions on imaging examinations (e.g., chest CT or X-ray) with no residual lesions; (2) complete resolution of disease-related symptoms; and (3) no evidence of further recurrence or progression, with no need for continued targeted medical or surgical treatment.

Improvement rate referred to the proportion of discharged patients who showed improvement during the reporting period. This indicator was used to complement the limitations of cure rate, particularly for chronic diseases, and reflected short-term therapeutic efficacy. Improvement was defined as marked alleviation of symptoms but without complete resolution, or partial functional recovery without full restoration, as determined by clinical physicians and recorded on the medical record homepage at discharge. General criteria included: (1) reduction in lesion size or decrease in density on imaging, with residual lesions showing benign characteristics; (2) significant reduction in clinical symptoms; and (3) continued need for follow-up observation (e.g., imaging reexamination at 3 or 6 months) to assess further changes.

Average length of stay referred to the average number of hospital days per discharged patient, calculated as the total bed days occupied by discharged patients with a given disease category divided by the number of discharged patients with that disease category during the reporting period. Bed day data were extracted from the HIS bed occupancy records.

Average cost per case was a cost-monitoring indicator used to evaluate the economic efficiency and cost-control level of medical resource utilization, reflecting the average economic cost per inpatient episode. It was calculated as the total hospitalization cost for a given disease category during the statistical period divided by the total number of discharged patients with that disease category during the same period. Hospitalization costs included expenses incurred during inpatient treatment, such as medication, treatment procedures, bed charges, and nursing care, all of which were extracted from the HIS cost records. Notably, medication costs were influenced by national bulk-buy drug procurement policies, which had been implemented prior to the study period (2022–2023). The relevant policy environment remained consistent across both years, thereby minimizing its confounding effect on the comparison of average cost per case between the two periods. The primary direct factors influencing average cost per case were thus medical factors, including treatment modalities, length of stay, diagnostic and laboratory tests, and other procedure-related variables.

### Statistical methods

All statistical analyses were performed using SPSS 23.0 (IBM Corp., Armonk, NY, USA). Descriptive statistical methods were applied, and count data were presented as numbers (%). Measurement data are presented as mean (SD) and conformed to normal distribution. An independent samples *t*-test was used to compare the mean values of average length of stay and average cost per case for each disease category between 2022 (control group) and 2023 (experimental group), and p-values were calculated. Cohen’s d was calculated using the number of discharged patients in 2022 and 2023 for each disease category as the sample size.

## Results

### Medical resource utilization

After implementation of the medical–rehabilitation integration model, all indicators of hospital medical resource utilization demonstrated varying degrees of improvement. The bed occupancy rate increased from 78.5 % before implementation to 86.2 %, representing a 9.8 % increase, indicating more effective utilization of bed resources through optimization of diagnostic and therapeutic processes and acceleration of patient recovery and discharge. The bed turnover rate increased from 28.7 to 34.1 times per year, corresponding to an 18.8 % increase, further reflecting improved bed turnover efficiency. The average length of stay decreased from 11.4 to 10.3 days, a reduction of 9.6 %, indicating a notable shortening of hospitalization duration. This improvement was partly attributable to enhanced diagnostic and treatment efficiency achieved through multidisciplinary collaboration, early rehabilitation intervention, and information sharing.

The utilization rate of major rehabilitation equipment increased from 62.4 to 75.9 %, reflecting a 21.6 % increase, indicating more coordinated allocation and use of rehabilitation equipment under the medical–rehabilitation integration model, with substantial reductions in equipment idleness and repetitive utilization. The average workload per medical staff member increased from 48 to 54 cases per person, corresponding to a 12.5 % increase, which reflected growth in patient volume and rehabilitation demand. However, no resource waste was observed under optimized workflows and clearly defined divisions of labor. The human resource allocation efficiency score increased from 70.2 to 78.4 points, an improvement of 11.7 %, indicating effective optimization of medical team deployment and collaboration within the medical–rehabilitation integration framework (Table 2).

**Table 2:** Comparison of medical resource utilization indicators before and after implementation of the medical–rehabilitation integration model at Nantong Rehabilitation Hospital.

Indicator	Before implementation	After implementation	Change rate
Bed occupancy rate, %	78.5	86.2	+9.8
Bed turnover rate (times/year)	28.7	34.1	+18.8
Average length of stay (days)	11.4	10.3	−9.6
Utilization rate of major rehabilitation equipment, %	62.4	75.9	+21.6
Average workload per medical staff member (cases/person)	48	54	+12.5
Human resource allocation efficiency score (points)	70.2	78.4	+11.7

### Operational efficiency of major disease categories

Due to variations in technical capacity, disease characteristics, and availability of medical resources, medical–rehabilitation integrated treatment was implemented across 18 clinical departments, accounting for 72 % of all hospital departments, thereby exerting a measurable impact on overall institutional operation and development. To further evaluate the effectiveness of medical–rehabilitation integration within specific disease categories, conditions with the highest discharge volumes in 2023, (the year of model implementation) were selected. These conditions included coronary atherosclerotic heart disease, hemiplegia, cerebral infarction, type 2 diabetes, chronic obstructive pulmonary disease (COPD), benign prostatic hyperplasia, and rib fractures. Key operational efficiency indicators, including average length of stay and average cost per case, were compared between periods before and after implementation of the medical–rehabilitation integration service model to assess its practical impact (Table 3).

**Table 3:** Comparison of operational efficiency indicators for major disease categories before and after implementation of the medical–rehabilitation integration model.

Disease category	Year	Number of discharged patients	Cure rate, %	Improvement rate, %	Average length of stay (days)	Average cost per case (RMB)
Coronary atherosclerotic heart disease	2022	958	------	98.8	12	12,074
Hemiplegia	2023	1,189	------	99.4	11	11,426
Cerebral infarction	2022	464	------	98.1	27	18,516
Type 2 diabetic peripheral neuropathy	2023	554	------	99.8	24	17,545
Chronic obstructive pulmonary disease	2022	464	------	97.4	11	15,549
Benign prostatic hyperplasia	2023	510	------	98.1	10	13,213
Rib fractures	2022	271	------	99.6	9	10,671
	2023	373	------	100	8.3	8,469
Chronic obstructive pulmonary disease	2022	274	------	97.1	14	16,619
	2023	338	------	97.6	13	15,758
Benign prostatic hyperplasia	2022	196	69.9	28.1	11	26,879
	2023	291	72.7	25.6	9	23,208
Rib fractures	2022	204	72.5	26.7	12	10,552
	2023	238	75.7	23.8	9	7,831

“------” indicates non-applicability for these conditions.

In 2023, discharge volumes increased across all disease categories, indicating enhanced institutional capacity for patient management following model adoption. With the exception of COPD, cure rates increased across all categories, with particularly notable improvements observed in hemiplegia and type 2 diabetes, for which cure rates approached 100 %. Slight reductions in improvement rates for benign prostatic hyperplasia and rib fractures were observed, possibly reflecting reclassification of cases previously categorized as improved into cured status following enhanced treatment outcomes. Average length of stay decreased across all disease categories, indicating improvements in treatment efficiency. In addition, average costs per case declined for all categories, indicating that the medical–rehabilitation integration model reduced medical costs while improving therapeutic effectiveness.

To objectively evaluate the practical effectiveness of the medical–rehabilitation integration model, verify its overall value in improving resource utilization efficiency and optimizing clinical pathways, and reveal its differential impacts across various disease populations – thereby providing empirical evidence for model optimization, resource allocation, and subsequent health policy formulation – statistical analyses were conducted on the core indicators of operational efficiency (average length of stay and average cost per case). The results are presented in Table 4.

**Table 4:** Statistical test results of core operational efficiency indicators.

Disease category	Indicator	2022 (control group)	2023 (experimental group)	p-Value	Effect size (Cohen’s d)
Coronary atherosclerotic heart disease	Average length of stay	12 days	11 days	0.012	0.23
	Average cost per case	RMB 12,074	RMB 11,426	0.008	0.19
Hemiplegia	Average length of stay	27 days	24 days	<0.001	0.47
	Average cost per case	RMB 18,516	RMB 17,545	<0.001	0.26
Cerebral infarction	Average length of stay	11 days	10 days	0.032	0.31
	Average cost per case	RMB 15,549	RMB 13,213	<0.001	0.38
Type 2 diabetic peripheral neuropathy	Average length of stay	9 days	8.3 days	<0.001	0.24
	Average cost per case	RMB 10,671	RMB 8,469	<0.001	0.41
Chronic obstructive pulmonary disease	Average length of stay	14 days	13 days	<0.001	0.36
	Average cost per case	RMB 16,619	RMB 15,758	<0.001	0.28
Benign prostatic hyperplasia	Average length of stay	11 days	9 days	<0.001	0.42
	Average cost per case	RMB 26,879	RMB 23,208	<0.001	0.46
Rib fractures	Average length of stay	12 days	9 days	<0.001	0.58
	Average cost per case	RMB 10,552	RMB 7,831	<0.001	0.63

Based on the table, the trends in length of stay and costs are as follows: average length of stay generally decreased (median: 11 days→9 days), with benign prostatic hyperplasia showing a reduction of 27 %; average cost per case decreased significantly (median: RMB 12,000→RMB 9,000), with rib fractures showing the largest reduction (27.2 %). From this analysis, following the implementation of the medical–rehabilitation integration model, hospitalization efficiency improved: the average length of stay across all disease categories decreased (Δ=1.7 days), and the average cost per case decreased (Δ=RMB 3,235).

## Discussion

### Core value of the medical–rehabilitation integration model in operational efficiency

The medical–rehabilitation integration model produced multidimensional improvements in operational efficiency at Nantong Rehabilitation Hospital. These improvements – particularly the reduction in average length of stay and increased utilization of rehabilitation equipment – alleviated persistent challenges related to resource underutilization and operational inefficiency that are commonly observed in rehabilitation settings. As shown in Table 2, the magnitude of these changes (ranging from 9.6 to 21.6 %) is clinically meaningful and comparable to those reported in enhanced recovery programs internationally [6], 7].

With respect to disease-category management, across seven high-frequency conditions, including coronary atherosclerotic heart disease and hemiplegia, concurrent improvements were observed in cure rates, along with reductions in length of stay and medical costs. The median average length of stay decreased from 11 to 9 days, and the median average cost per case declined from RMB 12,000 to RMB 9,000. These findings indicate that the medical–rehabilitation integration model was adaptable to diverse rehabilitation demands and demonstrated practical value in achieving cost control and efficiency enhancement, consistent with the functional positioning of tertiary rehabilitation hospitals as providers of specialized rehabilitation services with efficient care delivery.

### Pathway and existing shortcomings for developing the medical–rehabilitation integration model

Implementation of the medical–rehabilitation integration model in tertiary rehabilitation hospitals has generated experience with potential for replication. Development of this model has not relied on isolated adjustments but has followed a systematic pathway structured around four core dimensions, including organization, process, resources, and mechanisms. Each stage of implementation has been closely aligned with the central objective of removing barriers between medical treatment and rehabilitation and achieving coordinated, full-cycle service delivery, as reflected in the following areas.

(1) Multidisciplinary collaboration. The medical–rehabilitation integration model implemented by the hospital incorporated coordinated participation from multidisciplinary teams spanning medical, rehabilitation, and nursing services, thereby establishing an integrated and efficient collaborative framework. Specialists in internal medicine, surgery, and related fields provided specialized diagnostic and therapeutic support, while the rehabilitation department assumed a central role in medical–rehabilitation integration [2]. Rehabilitation physicians led the formulation of individualized rehabilitation programs, with rehabilitation therapists and nursing staff responsible for precise implementation and continuous service delivery. This collaborative approach demonstrated clear advantages in the comprehensive management of diverse disease categories, particularly among patients with complex clinical conditions [8]. By reducing disciplinary boundaries, close alignment between clinical treatment and rehabilitation was achieved, enabling the delivery of integrated care encompassing both disease management and functional recovery, with corresponding improvements in rehabilitation outcomes and patient experience.
(2) Personalized solutions. The medical–rehabilitation integration model reduced the conventional separation between medical treatment and rehabilitation, enabling close collaboration and organic alignment between the two and strengthening the coherence and systematic nature of healthcare services [9]. Emphasis was placed on individualized rehabilitation planning, with precise and feasible diagnostic and therapeutic strategies formulated according to patients’ clinical conditions and stage-specific recovery responses. This approach supported the attainment of predefined therapeutic goals, shortened the rehabilitation course, and promoted earlier reintegration into daily life and society, reflecting a patient-centered service orientation.
(3) Early rehabilitation intervention. Policy guidance from the World Health Organization on rehabilitation within health service systems recommends the incorporation of rehabilitation services across the continuum of care, particularly within tertiary medical institutions. Enhanced awareness of early rehabilitation intervention and improvements in its timeliness and effectiveness are recognized as key indicators for evaluating rehabilitation quality. Rehabilitation interventions were initiated during the acute treatment phase, and early rehabilitation has been demonstrated to reduce complications, promote functional recovery, shorten hospitalization duration, and improve overall rehabilitation outcomes [10], 11].
(4) Diverse service content. Service provision extended beyond physical and exercise-based rehabilitation to include integrated interventions addressing mental health, training in activities of daily living, and nutritional guidance. This diversified model overcame the limitations of single-function rehabilitation by delivering comprehensive care encompassing physical function restoration, psychological adjustment, and the redevelopment of self-care capacity. As a result, systematic management of overall health was achieved, diverse rehabilitation needs were addressed, and patients were supported in attaining functional independence and improved health status.
(5) Support from information technology construction. An integrated information-sharing and management platform was established to enable standardized and intelligent management of medical records, rehabilitation progress, and follow-up treatment recommendations. This infrastructure supported full-cycle management requirements under the medical–rehabilitation integration model, ensured effective coordination between clinical treatment and rehabilitation, and enhanced the overall rehabilitation experience for patients.
(6) Support from talent development. A foundational talent pool centered on specialized rehabilitation physicians and therapists was established. Team members completed long-term, structured rehabilitation training and demonstrated strong theoretical expertise and advanced practical skills, providing a solid basis for the delivery of high-quality rehabilitation services. In addition, continuous optimization of incentive policies and compensation guarantee mechanisms was undertaken to strengthen institutional attractiveness and the retention of high-level rehabilitation professionals. These advances in talent development reinforced core institutional capacity for multidisciplinary integrated diagnosis and treatment and comprehensive rehabilitation services, providing sustained support for ongoing improvement in service quality and for meeting the diverse rehabilitation needs of patients.

Despite these advances, several shortcomings remain within the current model. A primary limitation involves inadequate service continuity, which represents one of the most fundamental and prevalent challenges. Evidence indicates that rehabilitation services in major tertiary rehabilitation hospitals in China remain predominantly concentrated in the inpatient diagnostic and treatment phase. The absence of standardized referral pathways linking in-hospital rehabilitation during the acute phase with community-based or home-based rehabilitation during the recovery phase has resulted in fragmentation across care settings. Consequently, sustained rehabilitation intervention for patients with chronic conditions after discharge is often insufficient, limiting the continuation of functional training in daily life, increasing the risk of relapse, and reducing long-term rehabilitation effectiveness.

In addition, integration among clinical practice, education, and research remains limited. Systematic investigation of rehabilitation technology innovation and chronic disease rehabilitation mechanisms has been insufficient, with research activities characterized by fragmentation and weak linkages with universities and general hospitals. This has contributed to a limited number of high-quality research outputs and delayed translation of findings. Currently, internationally, rehabilitation robotics technology has become a key development direction in the field of stroke rehabilitation [12]. At the educational level, heavy clinical workloads have constrained clinicians’ capacity to participate in teaching activities, resulting in inconsistencies in teaching quality. Policy support for research funding applications and teaching qualification certification has also remained inadequate. Furthermore, insufficient long-term strategic planning and resource allocation for education and research within hospitals have impeded the fulfillment of the integrated clinical, research, and teaching functions expected of tertiary institutions.

Finally, policy and payment protection mechanisms remain underdeveloped. Health insurance coverage for rehabilitation services has been limited, with restricted reimbursement scopes and low reimbursement rates imposing substantial financial burdens on patients and hindering completion of the full rehabilitation trajectory. At the same time, the absence of unified evaluation standards for medical–rehabilitation integration services has affected objective assessment and effective quality supervision.

### Optimization suggestions for developing a medical–rehabilitation integration model

First, strengthening the three-tier rehabilitation service chain is recommended. Rehabilitation represents a continuous process encompassing the acute phase, recovery phase, and long-term rehabilitation. Accordingly, optimization of the medical–rehabilitation integration model in tertiary rehabilitation hospitals should ensure provision of systematic support from admission through discharge and into the home-based rehabilitation stage. A hospital–community collaborative framework should be established through formal cooperation agreements with surrounding community health service centers [13]. Within this framework, hospitals may provide rehabilitation assessment equipment and technical training, while guiding community physicians in the development of individualized rehabilitation prescriptions for chronic diseases and avoiding standardized, non-tailored service packages. For example, advancements in treatment technology have enabled the application of remote monitoring and artificial intelligence in cardiac rehabilitation [14].

Flexible follow-up strategies should be designed based on rehabilitation stage and individual variation, with rehabilitation progress monitored regularly through telephone, video, and related modalities. Rehabilitation plans should be adjusted in a timely manner to maintain continuity of therapeutic benefit. In addition, in-hospital rehabilitation data should be synchronized through information-sharing platforms to ensure consistency between community-based rehabilitation plans and hospital treatment protocols. Remote rehabilitation systems may serve as efficient and accessible tools for the delivery of home-based stroke rehabilitation, with demonstrated benefits for physical recovery as well as psychological and social well-being [14].

Furthermore, extension of home rehabilitation services is advised, including the establishment of home service teams to provide post-discharge rehabilitation guidance and recommendations for home environment modification. Development of an online rehabilitation guidance application, incorporating individualized training videos and monitoring scales, may further support adherence to home-based rehabilitation and facilitate remote clinical follow-up, thereby reducing the risk of recurrence.

Second, enhancement of the collaborative capacity of clinical practice, education, and research is recommended. Within the medical–rehabilitation integration model, tertiary rehabilitation hospitals should prioritize the development of advanced research systems and the optimization of teaching and talent training models to support sustainable institutional growth. In the area of scientific research, collaborative innovation should be actively promoted through the joint establishment of high-level rehabilitation medicine research institutes with university programs in rehabilitation medicine and relevant departments of general hospitals, enabling more systematic and applied research [15]. Support for applications to rehabilitation-related research projects at all levels should be strengthened, mechanisms for the efficient translation of research outcomes should be established, and dedicated units for intellectual property management and technology transfer should be created. These measures can facilitate the transformation of routinely accumulated clinical case resources into high-value research outputs, thereby enhancing institutional research capacity, innovation performance, and academic influence.

In the domain of education and talent development, implementation of a structured mentorship system with individualized guidance from senior rehabilitation physicians and therapists is recommended, together with reinforcement of practical skills training through the simulation of common clinical rehabilitation scenarios. Applications for teaching accreditation from higher medical institutions may also be pursued to support clinical internships and standardized training programs, thereby fulfilling responsibilities for rehabilitation education and the cultivation of interdisciplinary professionals. Through an integrated development strategy encompassing research and education, hospital core competitiveness may be strengthened, supporting the effective advancement of medical–rehabilitation integration objectives.

Third, strengthening policy support and resource guarantee mechanisms is essential. International rehabilitation policy frameworks encompass six core domains, including leadership and governance, financing, human resources, service delivery, rehabilitation-related medical technologies, and rehabilitation and health information systems. Within China, implementation of the medical–rehabilitation integration model in tertiary rehabilitation hospitals remains highly dependent on policy guidance and resource support. Coordinated engagement among government, hospitals, and society is required to secure financial investment, equipment provision, and qualified personnel, thereby supporting the long-term development of rehabilitation services [16].

In particular, support through medical insurance policy is critical. Medical insurance reimbursement functions as an important regulatory instrument for guiding healthcare delivery behavior and resource allocation, while also serving as a key incentive mechanism for promoting integration of medical and health services. Given the prolonged treatment cycles and relatively high costs associated with rehabilitation care, patients requiring long-term intervention experience a substantial financial burden. The establishment of a scientifically grounded and equitable medical insurance support system therefore represents a foundational requirement for stable expansion of rehabilitation services.

Hospitals should actively participate in policy formulation and feedback processes by providing empirical evidence derived from clinical practice to enhance policy feasibility and effectiveness. At the same time, rigorous and transparent systems for quality supervision and cost control in rehabilitation services should be implemented. Through standardization of clinical pathways and reinforcement of rational fee oversight, inappropriate charging practices and overtreatment may be effectively prevented. These measures contribute to improved efficiency in the utilization of medical insurance funds while safeguarding service quality and patient safety, thereby supporting balanced advancement of medical insurance sustainability and the healthy development of medical–rehabilitation integration.

### Limitations

Several methodological limitations should be considered. First, this was a retrospective, single-center study with a pre-post design using historical controls. While appropriate for exploratory analysis, this design cannot fully account for temporal trends or unmeasured confounders; thus, findings should be interpreted as associations rather than causal effects. Second, patient-level characteristics such as comorbidity burden and disease severity were not adjusted for due to data constraints, which may introduce selection bias. Third, patient-reported outcomes (e.g., quality of life, functional independence) were not collected. Fourth, the single-center nature limits generalizability. Fifth, the one-year follow-up period is relatively brief. Sixth, regarding outcome validation, cure and improvement determinations were made by attending physicians based on disease-specific criteria and recorded on medical record homepages. While the hospital implemented routine medical record quality control, no formal inter-rater reliability testing was performed for cure/improvement classifications. This should be addressed in future prospective studies. Future prospective multicenter studies with longer follow-up and patient-centered outcomes are needed to further validate the findings.

## Conclusions

Through an empirical evaluation of a medical–rehabilitation integration model implemented in a tertiary rehabilitation hospital in Nantong city, the effects of this model on medical service delivery, medical resource utilization, and the operational efficiency of major disease categories were examined. The findings indicated that the integration of acute-phase management, early rehabilitation intervention, rehabilitation-phase intervention, and long-term management enabled continuity across all stages of diagnosis and treatment, thereby addressing patients’ comprehensive and individualized needs related to diagnosis, treatment, and rehabilitation. After implementation of the model, notable improvements were observed in bed occupancy rate, bed turnover rate, utilization of major rehabilitation equipment, and medical staff workload. In addition, average length of stay was reduced, cure and improvement rates for major disease categories increased, and average cost per case declined.

The advantages of the medical–rehabilitation integration model were further reflected in strengthened multidisciplinary collaboration, earlier rehabilitation intervention, application of information technology support, and refinement of clinical and management processes. On this basis, recommendations were proposed to strengthen the three-tier rehabilitation service chain, enhance the integrated development of clinical practice, education, and research, and secure sustained policy and resource support to promote the long-term development of the medical–rehabilitation integration model.

Current academic research in this field has been progressing toward deeper multidisciplinary collaboration, broader application of information technologies, optimization of individualized and precise rehabilitation strategies, and reinforcement of early rehabilitation intervention. Concurrent emphasis has been placed on innovation in service delivery models, stronger linkage with community-based and home-based rehabilitation, refinement of policy and payment systems, and coordinated advancement of clinical practice, education, and research. Collectively, these efforts aim to establish a modern rehabilitation service system oriented toward the full care continuum, thereby improving the quality, efficiency, and accessibility of rehabilitation services. Future studies should employ prospective multicenter designs with larger sample sizes and longer follow-up periods to further validate the findings.
